# Applications of ancestral sequence reconstruction for understanding the evolution of plant specialized metabolism

**DOI:** 10.1098/rstb.2023.0348

**Published:** 2024-11-18

**Authors:** Todd J. Barkman

**Affiliations:** ^1^ Department of Biological Sciences, Western Michigan University, Kalamazoo, MI 49008, USA

**Keywords:** enzyme evolution, specialized metabolism, ancestral sequence reconstruction

## Abstract

Studies of enzymes in modern-day plants have documented the diversity of metabolic activities retained by species today but only provide limited insight into how those properties evolved. Ancestral sequence reconstruction (ASR) is an approach that provides statistical estimates of ancient plant enzyme sequences which can then be resurrected to test hypotheses about the evolution of catalytic activities and pathway assembly. Here, I review the insights that have been obtained using ASR to study plant metabolism and highlight important methodological aspects. Overall, studies of resurrected plant enzymes show that (i) exaptation is widespread such that even low or undetectable levels of ancestral activity with a substrate can later become the apparent primary activity of descendant enzymes, (ii) intramolecular epistasis may or may not limit evolutionary paths towards catalytic or substrate preference switches, and (iii) ancient pathway flux often differs from modern-day metabolic networks. These and other insights gained from ASR would not have been possible using only modern-day sequences. Future ASR studies characterizing entire ancestral metabolic networks as well as those that link ancient structures with enzymatic properties should continue to provide novel insights into how the chemical diversity of plants evolved.

This article is part of the theme issue ‘The evolution of plant metabolism’.

## 1. Introduction

Specialized metabolites are a diverse set of molecules that serve a wide array of functions in plant biology [1]. Well documented roles include not only signalling for defence against pathogens and herbivores but also the attraction of beneficial mutualists such as pollinators [2]. Hormone modulation, UV protection and osmoregulation are also achieved by specialized metabolite formation [3]. Their biosynthesis is fascinating, with enzymes that can generate multiple products from a single substrate, such as terpene synthases [4], as well as those that can catalyse reactions with multiple substrates to produce an array of different products, such as acyltransferases [5]. There is also growing evidence that the same specialized chemical can be synthesized by unrelated enzymes using different chemistry [6,7] as well as a surprising number of reactions that appear to be non-enzymatic/spontaneous [8,9]. One hallmark of specialized metabolism is the sporadic distribution of many metabolites, which shows that entire pathways can evolve independently and repeatedly using homologous or unrelated sets of enzymes [10]. Some general principles for the evolution of specialized metabolism have emerged, including that many pathways appear to have origins from primary metabolism [3,11]. Recruitment of divergent enzymes into specialized metabolic pathways results from gene duplication of the primary metabolic enzymes, which frequently become organized with others into operon-like clusters [12]. Various ‘omics’ techniques have accelerated great progress towards deciphering the modern-day genetic underpinnings of specialized metabolite biosynthesis and how the vast diversity of specialized metabolism has evolved [1,3]. Yet, further insight will likely require the use of more historically explicit approaches like ancestral sequence reconstruction (ASR) combined with experimental characterization.

ASR involves the estimation of extinct protein sequences followed by their resurrection using molecular biology techniques [13–16]. The activities of such resurrected molecules can then be experimentally determined and directly compared with what is known for their modern-day descendants to understand functional change over time. Because numerous reviews have been published in the last 10 years [14,17–20], only a cursory summary of the approach to ASR is provided here. ASR proceeds by the generation of an alignment of homologous sequences from which an optimal tree is estimated, assuming a model of nucleotide/amino acid substitution. The empirical Bayes approach is the most widely used method for ancestral sequence prediction and makes use of the branching pattern, branch lengths, alignment and substitution model to provide some measure of uncertainty associated with the estimated state for each aligned position at each node of the tree [21,22]. Once a sequence estimate is obtained, gene synthesis usually includes codon optimization for the host organism to be used for heterologous protein expression. Simulation studies show that all methods are reasonable but that maximum likelihood (ML) approaches provide the highest accuracy, especially compared with parsimony [23–25]. All methods assume the alignment and tree are known without error [26]. Nonetheless, simulation studies have suggested that errors in the branching structure and branch lengths may have a minimal impact on the estimates of ancestral sequences in many cases [25,27,28], but accuracy also depends on how much phylogenetic signal exists in the dataset [29] and rarely considered phenomena such as recombination [30]. Reconciling gene trees to use species tree-aware methods may also improve accuracy [31]. Estimating positions in an ancestral sequence is more complicated when there are alignment gaps [32–34]. Usually, parsimony is used to estimate whether an ancestral position was present or not, but ML methods can also be applied [22]. Alignment gaps are often particularly abundant at the N- and C-termini, where length variation due to indels is commonly observed. If the N-terminal portion of a protein encodes a signal peptide, which may be poorly conserved among divergent species, ASR may proceed by excluding those regions since they are not necessary for many heterologous protein expression systems. Whereas proteins from primary metabolism may be easily aligned and allow highly confident ASR, not all proteins from a highly diversified specialized metabolite enzyme family are equally amenable to ASR. Sadly, there is no metric that can indicate whether ASR will be reliable for any particular protein alignment, but it is generally true that higher posterior probabilities are associated with sequence estimates from more recent divergences.

## 2. Interrogation of the plant tree of life using ancestral sequence reconstruction is in its infancy

**Figure 1.**
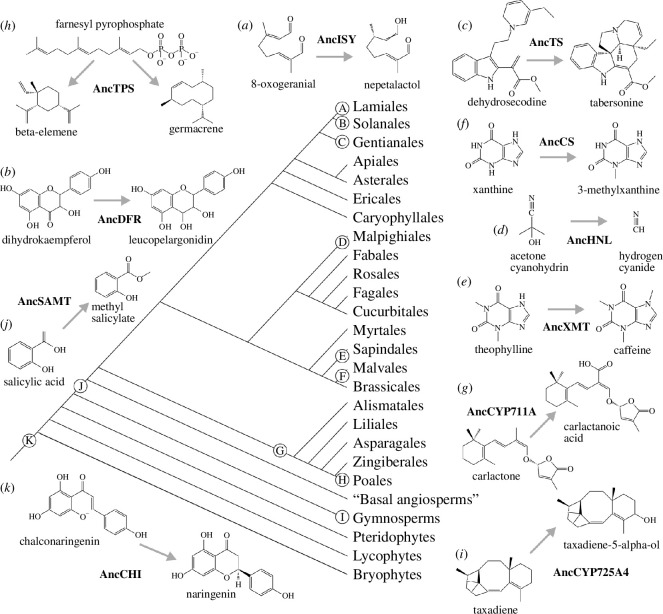
A land plant phylogeny shows the lineage-specific context of published studies using ancestral sequence reconstruction to understand the evolution of plant specialized metabolism. Labelled circles show the primary lineages investigated; however, while some studies investigated multiple nodes in plant phylogeny, those are not shown for the sake of clarity. Biosynthetic reactions are labelled to correspond to branches along which ancient proteins were resurrected. In many cases, additional reactions or substrate stereoisomers were studied but only exemplars are shown.

ASR has found a place in studies of plant proteins, with ribulose-1,5-bisphosphate carboxylase/oxygenase (Rubisco) being a prominent target given its importance for planetary carbon fixation and interesting kinetics with CO_2_ and O_2_ [35–37]. Additional ASR studies of non-enzymatic proteins include light sensing in ancient cyanobacteriochromes [38], ancestral interactions of MADS box [39] and cyanobacterial photoprotection proteins [40], self-incompatibility ligand–receptor evolution [41] and microRNA effects on plant development [42]. Because ASR is a relatively new technique, there have been only 11 applications to study aspects of ancient plant specialized metabolism (figure 1) [8,43–54]. Most studies have focused on the more recent origins of metabolic innovations that are often restricted to one or a few lineages and are likely less than 10 Ma old, such as terpene derivatives in Lamiales (figure 1*a*) and anthocyanins in Solanales (figure 1*b*). Others have targeted the ancient-most origins for metabolites such as methyl salicylate and flavonoids that are shared by most land plants and are more than 100 Ma old (figure 1*j,k*). Several classes of specialized metabolites have been studied, including terpenoids (figure 1*a,c*,*g*,*h*,*i*), alkaloids (figure 1*c,e,f*), flavonoids (figure 1*b,k*), benzenoids (figure 1*j*) and nitriles (figure 1*d*). The resurrected ancient enzymes perform various biochemical modifications of specialized metabolite substrates and include reductases (figure 1*a,b*), methyltransferases (figure 1*e,f,j*), cytochrome P450 oxidases (figure 1*g,i*), isomerases (figure 1*k*), lyases (figure 1*d*) and cyclases (figure 1*c,h*). ASR has found growing application as a tool for protein engineering, and plant metabolic enzymes are no exception. The study of an ancestral CYP725A4 taxadiene hydroxylase (figure 1*i*) was largely undertaken in order to improve enzymes for industrial purposes. Likewise, an ancestral photodecarboxylase from *Chlorella* and relatives exhibits properties that could be useful for industry [55]. In spite of the progress made, abundant lineages remain unstudied and many of these are well known for producing some of the most iconic specialized plant metabolites, such as glucosinolates, largely restricted to Brassicales, and betalains, found in Caryophyllales. Of course, since the biochemical basis for the production of many metabolites in modern-day species remains unknown, it is hardly a surprise that studies using ASR have yet to be conducted! Below we discuss these ASR studies in terms of the implications of their findings for understanding specialized metabolite evolution.

## 3. Catalytic proteins may emerge from inactive ancestors to produce specialized metabolites

One study on the origins of plant specialized metabolic enzymes is particularly notable because it showed how a catalytic enzyme arose from a non-catalytic ancestor [49]. Chalcone isomerase (CHI) is a crucial early-pathway enzyme providing flux to various pigments and other flavonoid metabolites in all land plants (figure 1*k*). CHI is homologous to fatty acid-binding proteins that are non-catalytic in a wide array of organisms. To understand how CHI activity evolved, Kaltenbach *et al*. [49], used ASR to assay the progenitor proteins that gave rise to the catalytic enzyme. They found that an ancient non-catalytic ancestor, presumably involved in fatty acid binding, was duplicated in early land plants, and one of its descendants evolved enzymatic activity to give rise to CHI in modern-day plants. The initial stages of enzymatic emergence appear to have required an increase in the size of the active site to allow for productive chalconarigenin substrate binding because the active site of the non-catalytic ancestor was smaller, such that only non-productive binding would likely occur [49]. Ultimately, it appears that one of the most important catalytic residues for CHI activity was actually found in the ancestral, non-enzymatic fatty acid-binding protein (FABP)-like protein but only later did it become repositioned by other mutations to allow the emergence of enzymatic activity. The evolutionary trajectory towards catalytic activity did not involve strong negative epistatic mutations and, surprisingly, could have evolved via multiple different mutational paths—even some involving mutations that did not occur historically [49]. This study stands as an interesting example of enzyme activity evolving without having a promiscuous ancestor, and this catalytic innovation appears to have been concomitant with early land plant adaptation. It is not clear how many specialized metabolic enzymes have arisen from non-catalytic ancestral proteins, but catalytic promiscuity/plasticity is known in many modern-day enzymes like fatty acid desaturases [56]; therefore, ASR can likely also improve our understanding of the origins and shifts of catalytic properties more broadly.

## 4. Apparent exaptations of promiscuous ancestral enzymes seem to promote the evolution of novel descendants

Many models of protein functional evolution require gene duplication and posit the existence of multifunctional/promiscuous ancestors [57–61]. The multiple different ancestral activities, which may be thought of as exaptations, can then be partitioned by descendants after gene duplication, thereby contributing to functional diversification [62–65]. The assumption of promiscuous ancestors can be explicitly tested using ASR [66], and several studies of plant specialized metabolism have done just that. One such study system involves the enzymatic generation of hydrogen cyanide (HCN), which has evolved in numerous independent angiosperm lineages and has a potent role in herbivore defence. In the well studied cyanogenic members of Euphorbiaceae (e.g. cassava), the hydroxynitrile lyase (HNL) enzymes that generate HCN (figure 1*d*) have been identified, and they are most closely related to esterases that work on a variety of substrates including methyl salicylate to form salicylic acid during SAR for pathogen defence. Phylogenetic analysis suggests that HNL evolved from ancestral esterases, which prompts the question of how a different catalytic reaction could have arisen. To investigate this, Devamani *et al*. [50] and Rauerdink *et al*. [51] used ASR to reconstruct the ancient enzymes that would have given rise to the modern-day HNL [50,51]. In this case, it was shown that, although modern-day esterases are largely specific for ester cleavage, the ancestral enzymes could perform both ester hydrolysis and the elimination reaction to form hydroxynitriles. After gene duplication, one descendant lineage of proteins appears to have specialized as esterases and the other for hydroxynitrile lyase activity. Interestingly, there appears to be an evolutionary incompatibility between specialization for lyase or esterase activity because mutations to improve one activity came at the expense of the other. The structural basis for ancestral promiscuity of ancHNL1 was determined using X-ray crystallography and appears to be due to larger active site volume, which allows different binding orientations of each of the different classes of substrates [67].

In addition to catalytic promiscuity, substrate promiscuity has been reported for numerous ancestral specialized metabolic enzymes. One prominent example was documented from Lamiaceae, in which the origin of nepetalactone iridoids from catnip was studied (figure 1*a*) [48]. The origin of nepetalactones in catnip is particularly intriguing since iridoids appear to have been historically lost in much of the family, only to re-evolve within *Nepeta*. Iridoid synthase (ISY) is found to catalyse the formation of nepetalactol from 8-oxogeranial (8OH) in *Nepeta* to provide precursors for nepetalactone production. ISY is most closely related to progesterone reductase (PRISE), which contributes to the biosynthesis of cardenolides and other specialized metabolites in many plants. Lichman *et al*. [48] resurrected the ancestral PRISE that appears to have given rise to ISY in modern-day *Nepeta* to produce iridoid terpenoids. The progenitor was promiscuous and showed activity with both progesterone and 8OH. After gene duplication, one daughter enzyme appears to have specialized for progesterone reduction while the other evolved to specialize for activity with 8OH to lead to nepetalactol formation. Thus, ancestral substrate promiscuity promoted the re-evolution of iridoids in the catnip lineage. Substrate promiscuity is also known for several other lineages of enzymes, including ancestral methyltransferases producing volatile methyl esters [43]. In addition, cyclases involved in terpenoid production [8,53] demonstrate promiscuous activities to generate multiple products from a single substrate (figure 1*c,h*). Because so many modern-day specialized metabolic enzymes are either catalytically or substrate promiscuous [68], this finding for ancestral enzymes is hardly surprising. However, it is important to note that the promiscuous ancestral activities that we assume to be historically neutral exaptations may instead be biochemical adaptations with some selective value. To discern between the two possibilities is difficult because we cannot unambiguously determine whether the *in vitro* measured enzymatic activity actually had fitness value in the context of ancient plant physiology. Enzyme promiscuity has been leveraged in studies of protein engineering, thereby providing further evidence for the importance of this characteristic for generating novelty [69]. While the existence of ancestral promiscuity has been hypothesized to be involved in the generation of novel plant specialized metabolites, ASR has not only verified its role but allowed insights into the mechanisms by which it evolves.

## 5. Epistasis may or may not limit evolutionary transitions of plant specialized metabolic enzymes

Although epistasis is well known as a classical genetic phenomenon, studies of intramolecular epistasis are relatively new [70]. Positive or negative intramolecular epistatic interactions have been documented whereby one mutation may affect either the sign or magnitude of a phenotype caused by another mutation [71]. In the case of strong negative epistasis, evolutionary paths through an adaptive landscape may be constrained [72–76]. Smith *et al*. [47] investigated the impact of intramolecular epistasis on an anthocyanin biosynthetic colour transition in Solanaceae (figure 1*b*). Anthocyanins are a subnetwork of the flavonoid pathway and are notable for imparting red, purple and blue coloration to flowers, especially in the Iochrominae, which exhibit myriad colours with many inferred shifts between them. In a particularly integrative and sophisticated analysis, Smith & Rausher [77] mapped changes in floral pigmentation to three loci: flavonoid 3′-hydroxylase (F3′H), flavonoid 3′,5′-hydroxylase (F3′5′H) and dihydroflavonol reductase (Dfr). In red-flowered species, both F3′H and F3′5′H were either downregulated or pseudogenized to reduce flux to blue pigment precursors and increase it towards the red-pigment branch of the metabolic network. To study the impact of changes in Dfr (figure 1*b*), Smith *et al.* [47] resurrected ancestral Dfr from the blue-flowered ancestor and that of the red-flowered ancestor that gave rise to the modern-day red-flowered species *Iochroma gesnerioides* and *Iochroma fuchsioides*. The blue-flowered ancestral enzyme had a nearly equal preference for all three anthocyanin precursors, dihydrokaempferol (DHK), dihydroquercetin (DHQ) and dihydromyricetin (DHM), whereas the red-flowered ancestral enzyme had more than doubled its relative preference for the red-pigment precursor, DHK. To investigate the mutational basis for this shift in relative enzyme preference, Smith *et al*. [47] mutated three amino acid residues that differ between the two ancestral enzymes and made all possible combinations to evaluate potential epistasis. They found that there was statistically significant positive and negative epistasis among the three sites. The negative epistatic interactions are hypothesized to have restricted the evolutionary paths (order of substitutions) that could have occurred during the specialization for producing red anthocyanins. Whereas this and other studies have documented negative epistatic restrictions on evolutionary transitions [78,79], most studies of plant specialized metabolic enzymes show only minimal such effects.

For instance, negative epistasis was not detected for the origin of CHI activity (figure 1*k*) from a non-catalytic ancestral protein, thereby leading to the conclusion that evolution would not be limited [49]. More recently, it was shown that epistatic interactions also exist among mutations that underlie the origin of salicylic acid methylation preference by *S*-adenosyl-ʟ-methionine : salicylic acid carboxyl methyltransferase (SAMT) enzymes [45] (figure 1*j*). In this case, the evolution of novel methylation characteristics was mostly due to a single mutation. Although statistically significant intramolecular epistasis was detected, the interactions among mutations were of small positive magnitude such that evolutionary paths would not be restricted. The findings of these studies may be generally applicable since they are congruent with studies of modern-day plant specialized metabolic enzymes that have shown substrate preference switches may occur via one or a few substitutions [80]. Of course, if evolutionary transitions between any two proteins with different activities involve more than six or seven amino acid replacements, the number of mutants to generate and characterize is so vast as to preclude comprehensive analysis of epistasis owing to a lack of high-throughput techniques. However, a fruitful *tour de force* approach for the analysis of hundreds of mutated sesquiterpene synthases has been shown [81]. Future advancements in the study of intramolecular epistasis and its impact on the evolution of plant specialized metabolism will require robust statistical analysis [47,82]. New and existing statistics for epistasis should also be investigated in terms of their sensitivity to violations of assumptions as well as type I and type II error rates. Even in the face of statistical significance from *in vitro* studies, it remains to be seen how meaningful these intramolecular epistatic interactions may be in the context of a long-lived, multicellular plant. Whatever the case, if a particular order of mutations is required for enzymatic specialization, attempts to predict how to best engineer these kinds of enzymes for desired properties may be challenging [83].

## 6. Modern-day specialized metabolite pathways may evolve by sequential elaboration of ancestral pathway flux or by novel diversions

Modern-day plants exhibit pathways for specialized metabolite synthesis that are often catalysed by homologous enzymes borrowed from other pathways, including those of primary metabolism [12]. These enzymes are often promiscuous and this, coupled with the existence of non-enzymatic substrate conversions in many pathways, leads to the hypothesis that early metabolic networks would have produced an array of metabolites that might not be seen in modern-day plants [60,84]. One simple pathway that has been investigated using ASR is that of caffeine biosynthesis. Caffeine is biosynthetically derived from nucleotide metabolism and is found in flowers, where it can enhance pollinator memory [85], and in leaves, to provide herbivore defence [86]. In order to understand how the three terminal sequential methylation reactions of the pathway to caffeine evolved in the *Citrus*, *Theobroma* and *Paullinia* lineages, ancestral SABATH family methyltransferase enzymes were resurrected [44,46]. Since caffeine biosynthesis appears to employ either caffeine synthase (CS)-type or xanthine methyl transferase (XMT)-type enzymes (figure 1*e,f*), ASR was used on both paralogous groups of methyltransferases. In these three lineages, the multiple extant enzymes catalysing reactions in the modern-day caffeine pathways are inferred to have arisen from a single ancestral enzyme. In each case, the single ancestral enzyme was capable of performing multiple reactions within the xanthine alkaloid biosynthetic network to produce multiple different products. However, in no case was caffeine produced. Multistep ancient enzymes have been reported in other systems [52,87] and should be hypothesized to exist since earlier plants had less complex genomes to encode fewer enzymes to perform a comparable number of biosynthetic steps. Ultimately, independent gene duplication of the ancestral enzyme in each lineage gave rise to no fewer than two enzymes for the sequential methylation reactions towards caffeine. In each lineage, duplicated descendants became relatively more specialized to carry out different steps towards caffeine production. These results indicate that, at least for the final step of caffeine biosynthesis, this evolved last and built sequentially (or cumulatively) upon the reactions that had been put in place earlier. This model for pathway evolution assumes that the ancestral accumulation of metabolites would be selectively advantageous. Consistent with these assumptions, xanthine alkaloid precursors to caffeine can bind to adenosine receptors in rats and probably other mammals [88].

In a separate study, the origins of monoterpene indole alkaloid (MIA) biosynthesis in Apocynaceae species were investigated (figure 1*c*) [8]. MIAs are extremely diverse structures involved in plant defence and have a long history in traditional medicine but have now found a place in modern-day pharmaceuticals. The enzymes involved in aspidosperma- and iboga-type MIA biosynthesis in modern-day species appear to be derived from carboxylesterase-like cyclases and are classified as 2-hydroxyisoflavone dehydratase-like (HIDs) cyclase proteins. To understand how ancestral Apocynaceae species might have evolved to synthesize the current suite of MIAs of modern-day species, ancient HID-like enzymes were resurrected and assayed for activity with a central metabolic network precursor, angryline. Several of the ancestors appear to have been multistep enzymes that could channel products to more than one of the alkaloid pathways. In modern-day plants, the pathways towards three different types of MIAs do not sequentially build on ancestral products; rather, with each gene duplication, descendant enzymes evolved to divert flux from the same common ancestral precursor substrate, angryline, into either the aspidosperma- or the iboga-type alkaloid pathways [8]. Diversion of pathway flux after gene duplication has been hypothesized during the evolution of alpha-pyrone biosynthesis [89], making this a potentially more widespread phenomenon in plants. Specialized metabolite pathways are complex and may differ over developmental time as well as in different tissue types and in response to varying environmental stimuli; therefore, it is safe to say we are only at the beginning of our understanding of how pathways are evolutionarily assembled [87].

## 7. Embracing, and accounting for, uncertainty of ancestral sequence estimates

Of course, advancements towards understanding the evolution of plant specialized metabolism depend critically on the accuracy of ASR methods. However, because of the nature of the sequence estimation problem, there will always be uncertainty and any study using ASR should explicitly investigate the functional implications of it in some way [90]. A simple and frequently used approach to investigate uncertainty is to mutate a single site that has low posterior probability in the optimal sequence estimate and replace it with a suboptimal amino acid. Subsequently, the mutant and optimal protein can be compared in terms of enzymatic activity [43,70]. At the other end of the spectrum is the ‘AltAll’ approach, which advocates generating a second sequence in which the most likely amino acid is exchanged with the second-best amino acid at *every* site where the alternative is above a particular probability threshold (e.g. 0.2), followed by functional characterization to compare with the results obtained from the optimal estimate [91]. Another approach is to use multiple different datasets and compare the functions of the optimal sequence obtained from each. This might include using different alignments or models of protein substitution or comparing the results of a nucleotide and corresponding amino acid alignment; one advantage of using nucleotides is that ancestral codons may be estimated that encode amino acids not seen in any modern-day protein sequences. Ancestral sequences estimated by different statistical methods can also be compared [50,92]. Bayesian approaches are intuitively appealing since they explicitly incorporate variations of tree topology, branch lengths and model parameters into estimates [23,92,93] although their accuracy does not appear to exceed that of ML [25]. Methods with different assumptions, such as ProtASR [94], which strives to account for protein folding stability and how it might evolve over time, could be used to compare with traditional approaches. Other studies approach the question of uncertainty in the optimal protein sequence by generating a second set of ancestral estimates for the same nodes using different datasets. This could be achieved using differently curated alignments that only include genomic sequences or combine genome and transcriptome data. Also, owing to the rapidly increasing amount of data available every year, sequence estimates could change over time. Catania *et al*. [45] used a 40-fold larger dataset to investigate the uncertainty of ancestral amino acid sequence estimates obtained in a previous study [43] that had a different tree topology, branch lengths and amino acid substitution model [45]. In this particular case, the sequence identity for the alternative alleles for the same nodes ranged from only 75 to 83% but showed similar enzyme activity. In conclusion, there is no single best way to assess uncertainty in an ASR study and any approach to experimentally characterize the variation associated with a sequence estimate is better than ignoring its existence. The crucial point is that if experimentally determined enzyme assay results of multiple ancestral variants are comparable, then we will have higher confidence that the results are not artefactual or biased.

## 8. Future studies

ASR appears to be a valuable approach to inform studies of plant specialized metabolism. In particular, ASR has provided insight that is impossible to glean from the study of modern-day enzymes only. For instance, the required mutational order leading to enzyme functional divergence, bygone ancestral enzymatic activities and the role of promiscuity in evolution can only be guessed without ASR. Therefore, several long-standing questions regarding the evolution of plant specialized metabolism should continue to benefit from its application. One underexplored area of research is to resurrect entire ancestral metabolic networks to understand pathway flux evolution more broadly. This might require the resurrection and co-expression of numerous ancestral enzymes within a single cellular system or could be conducted *in vitro*. These studies will be important to enhance our understanding of how ancestral components could be integrated into a network in a patchwork fashion and to what extent underground metabolism is pervasive [12,84]. While studies of ancestral metabolism *in vitro* can provide some insight into the molecular evolutionary aspects of specialized pathways, systems that would allow selection using pathogens or herbivores on transgenic plants (or other model systems) are sorely needed. These would allow a better understanding of whether promiscuous enzyme activities or differing ancestral pathway fluxes have fitness consequences [95]. Finally, since there is evidence for the importance of metabolons for specialized metabolite formation [96], investigations using ASR could reveal how and why these are evolutionarily assembled.

## Data Availability

This article has no additional data.
